# Impact of Tryptophan Oxidation in Complementarity-Determining Regions of Two Monoclonal Antibodies on Structure-Function Characterized by Hydrogen-Deuterium Exchange Mass Spectrometry and Surface Plasmon Resonance

**DOI:** 10.1007/s11095-018-2545-8

**Published:** 2018-12-10

**Authors:** Tyler Hageman, Hui Wei, Patrick Kuehne, Jinmei Fu, Richard Ludwig, Li Tao, Anthony Leone, Marcel Zocher, Tapan K. Das

**Affiliations:** 1 Department of Chemistry, University of Kansas, 1567 Irving Hill Road, Lawrence, KS USA; 2 Biologics Development, Bristol-Myers Squibb, 311 Pennington Rocky Hill Road, Pennington, NJ 08534 USA

**Keywords:** Tryptophan oxidation, complementarity-determining region, monoclonal antibody, hydrogen-deuterium exchange mass spectrometry, surface plasmon resonance

## Abstract

**Purpose:**

Tryptophan’s (Trp) unique hydrophobic and structural properties make it an important antigen binding motif when positioned in complementarity-determining regions (CDRs) of monoclonal antibodies (mAbs). Oxidation of Trp residues within the CDR can deleteriously impact antigen binding, particularly if the CDR conformation is altered. The goal of this study was to evaluate the conformational and functional impact of Trp oxidation for two mAb subtypes, which is essential in determining the structure-function relationship and establishing appropriate analytical control strategies during protein therapeutics development.

**Methods:**

Selective Trp oxidation was induced by 2,2′-Azobis(2-amidinopropane) dihydrochloride (AAPH) treatment in the presence of free methionine (Met). The native and chemically oxidized mAbs were characterized by hydrogen-deuterium exchange mass spectrometry (HDX-MS) for conformational changes and surface plasmon resonance (SPR) for antigen-antibody binding.

**Results:**

Treatment of mAbs with AAPH selectively oxidized solvent accessible Trp residues. Oxidation of Trp within or in proximity of CDRs increased conformational flexibility in variable domains and disrupted antigen binding.

**Conclusions:**

Trp oxidation in CDRs can adversely impact mAbs’ conformation and antigen binding. Trp oxidation should be carefully evaluated as part of critical quality attribute assessments. Oxidation susceptible Trp should be closely monitored during process development for mAbs to establish appropriate analytical control for manufacturing of drug substance and drug product.

**Electronic supplementary material:**

The online version of this article (10.1007/s11095-018-2545-8) contains supplementary material, which is available to authorized users.

## Introduction

Monoclonal antibodies (mAbs) are a major class of protein therapeutics in drug development pipelines and in the biopharmaceutical drug market. To date, more than 60 mAbs have been approved for treating diseases in several therapeutic areas in the US (1). Currently, there are more than 50 mAbs in late-stage clinical studies and many more in early stages of development (2,3). One of the most valuable attributes of a therapeutic mAb is rooted in the selective binding to the antigen with high affinity via interaction of complementary determining regions (CDRs), which conveys sub-nanomolar potency (4,5).

Similar to other protein therapeutics, mAbs are susceptible to chemical and physical degradation during manufacture and storage (6,7). Impact on higher-order structure and biological function resulting from oxidation, isomerization, deamidation, and aggregation has been widely studied and reported (8–12). Depending on the location and extent of degradation, the safety, efficacy, and stability profiles could be altered.

A common chemical degradation pathway of protein therapeutics is oxidation. Oxidation can be induced by light exposure, thermal stress, or impurities such as metal ions and peroxides (13). Among all amino acid residues in mAbs, Methionine (Met) is the most susceptible residue to oxidation. The impact of Met oxidation on the structure-function relationship of mAbs has been studied extensively (14–18). Oxidation of other residues in proteins such as tryptophan (Trp), cysteine, phenylalanine, tyrosine, and histidine have also been reported (19–21). Aggregation, fragmentation, and loss of binding activity as a result of oxidation of these residues in mAbs have been reported while few have studied the impact on higher-order structure (22–26).

Trp residues are commonly found in CDRs due to their structural features and hydrophobic properties that confer niche recognition points that facilitate strong and specific binding between mAbs and their respective antigens. However, Trp in CDRs may be more susceptible to oxidation because of the relatively flexible and solvent accessible nature of CDR loops (27–29). Conserved Trp residues in the constant regions of mAbs are less solvent accessible, and hence less susceptible to oxidation as they are buried within the 3-D protein structure. Similar to exposed Met residues the exposure to radicals generated from metal, light, and thermal stress during mAb manufacture and storage increases the risk of exposed Trp oxidation (13,19). The mechanism of oxidation in relation to type of exposure directly influences the degree of oxidation risk. For example, exposed Met preferentially oxidizes in relation to exposed Trp under hydrogen peroxide exposure. In contrast, studies have demonstrated the preferential oxidation of Trp over Met under oxidizing conditions (25,26,30). Under oxidizing conditions, Trp can form various oxidation products including N-formylkynuernine (NFK), kynurenine (Kyn), hydroxytryptophan (OH-Trp), and 3-hydroxykynuerinine (3-OH-Kyn) (Fig. S1), increasing the complexity of analytical characterization (31). The hydrophobic nature of a Trp side chain is drastically changed once oxidized (32). Such changes can lead to undesirable changes of mAb attributes. Previously, it has been shown that oxidation of Trp residues in CDRs of mAbs can impact antigen-antibody binding activity (25,26). This is intuitive based on the increased polar nature and potential for hydrogen bonding for oxidized Trp residues. To date studies on Trp oxidation in CDRs have been limited to bioactivity and stability studies (13,23,25,26). A detailed understanding of the impact on the structure-function relationship in the context of the local and overall conformation of mAbs is still lacking. This knowledge is critical for identifying the critical quality attributes of mAbs currently in development in order to establish appropriate analytical control strategies for production and shelf life. It is also invaluable to future drug candidate screening and molecular design.

Hydrogen-deuterium exchange mass spectrometry (HDX-MS) is a powerful technique for characterizing local and global protein conformations in solution (33,34). Today the application of HDX-MS in development of biopharmaceuticals has substantially grown as the technique becomes more automated, powerful, and routine to use (35). HDX-MS has been widely used for the characterization of higher-order structure of mAbs (36,37). Multiple studies have been reported using HDX-MS to monitor the conformational impact of chemical and physical modifications (8,12,17,38). Others have used HDX-MS to understand the impact of chemical modifications on biological functions through conformational changes (9,18). HDX-MS results have revealed structural changes responsible for variation in function.

In this study we used HDX-MS to provide insight on the conformational impact of Trp oxidation in two mAbs of IgG1 and IgG4 subtypes. As a widely used technique for characterizing antibody-antigen binding activities, surface plasmon resonance (SPR) was used in addition to HDX-MS to gain a better understanding of the conformational impact of Trp oxidation on changes in function. To the best of our knowledge, this is the first report of using HDX-MS to study the conformational impact of Trp oxidation in the CDRs of mAbs.

However, it is challenging to study the structural and functional impact induced by Trp oxidation alone, since general stress conditions including UV light exposure, thermal stress, and oxidative agent exposure can induce Trp oxidation but also induce other modifications, such as aggregation, fragmentation, or methionine oxidation (39–41). To understand the structure-function impact of Trp oxidation in the CDRs, Trp residues need to be selectively oxidized to a significant extent without other major chemical/physical degradation. To achieve selective oxidation of susceptible Trp residues on mAbs, we used the approach of AAPH treatment in the presence of free Met (30). The oxidative mechanism of AAPH mimics that of radicals formed during irradiation and metal exposure (20,42). AAPH in combination with free Met minimizes Met oxidation and physical degradations while oxidizing susceptible Trp significantly under mild conditions and in a short timeframe.

With this approach, we selectively oxidized susceptible Trp to high levels on an IgG1 mAb with one susceptible Trp residue in the CDR, and on an IgG4 mAb with two susceptible Trp residues in the CDRs and one in the non-CDR of the variable domain. SPR analysis of the Trp oxidized material *versus* the reference material revealed that Trp oxidation in the CDR disrupted antigen binding for both mAbs, while only the localized regions exhibited increased flexibility as evident by HDX-MS analysis, indicating that the disruption of local structure contributed to decreased antigen binding. This study provides a general approach for exclusively evaluating the structure-function relationship between Trp oxidation in CDRs and its conformational impact on binding activities using selective AAPH treatment followed by HDX-MS and SPR analysis.

## Materials and Methods

### Materials

The IgG1 mAb (mAb1) and IgG4 mAb (mAb4) were produced in Bristol-Myers Squibb Company. They were expressed in Chinese hamster ovary (CHO) cells and purified by standard chromatographic steps. Both mAbs were frozen and stored at −80°C in formulation buffer. LC-MS grade water was purchased from Honeywell (Plainview, NY). LC-MS grade acetonitrile was purchased from J.T. Baker (Center Valley, PA). 8 M Guanidine-HCl, premium grade TCEP-HCl, and LC-MS grade formic acid were purchased from Thermo Scientific Pierce (Grand Island, NY). Sequencing grade trypsin was purchased from Promega (Madison, WI). Human Fab capture kit, CM5 Sensor Chip, HBS-EP+ running buffer and amine coupling kit was purchased from GE Healthcare Life Sciences (Piscataway, NJ). All other reagents were purchased from Sigma-Aldrich (St. Louis, MO) unless otherwise specified.

### Oxidized Sample Preparation

To selectively oxidize Trp residues, 2,2-azobis(2-amidinoporpane) dihydrochloride (AAPH) and L-methionine were directly dissolved in each mAb solution to achieve final concentrations of 25 mg/mL (AAPH), 33 mg/mL (L-methionine), and 15 mg/mL (mAb). Samples were incubated at room temperature protected from light. Oxidation reaction was stopped by buffer exchange with formulation buffer on an illustra NAP-5 column (GE Healthcare, Piscataway, NJ) and samples were stored at −80°C before being analyzed.

### Oxidized Sample Characterization

Tryptic peptide mapping with tandem mass spectrometry (MS/MS) was used to identify and quantify oxidation. Reference and oxidized samples were denatured, reduced, alkylated, and digested then separated in reversed phase liquid chromatography on a UPLC BEH C18 2.1 × 100 mm column (Waters, Milford, MA) with an Acquity UPLC H-Class system (Waters) and detected by a Q Exactive Plus mass spectrometer (Thermo Scientific, San Jose, CA). MS and MS/MS spectra were analyzed by Xcalibur (Thermo Scientific) to identify and quantify oxidation levels of tryptic peptides.

Size exclusion chromatography (SEC) was used to identify and quantify soluble aggregates from AAPH treatment. Reference and oxidized samples were separated on a TSKgel G3000SWxl 7.8 mm × 30 cm column (TOSOH Biosciences, King of Prussia, PA) on an Acquity UPLC H-Class system (Waters) with photodiode array detection at 220 nm. Absorbance traces were analyzed by Empower (Waters) to identify and quantify soluble aggregates.

### SEC Fractionation

SEC fractionation was used to separate and collect oxidized monomer from samples containing significant soluble aggregates. Aggregates and monomer species were separated on a TSKgel G3000SW 21.5 mm × 30 cm column (TOSOH Biosciences) and collected on an AKTA Avant 25 (GE Healthcare) fraction collector. Monomer fractions were combined, concentrated, and buffer exchanged with formulation buffer using an Amicon Ultra-15 centrifugal filter with a 30 kDa MWCO (EMD Millipore, Billerica, MA). Oxidized monomer sample was stored at −80°C before being analyzed.

### HDX-MS Protocol

HDX-MS experiments were performed on an HDX manager system with automatic sample handling, online digestion, and separation (Waters). Non-deuterated samples were prepared by diluting 3 μL (5 mg/mL) of each mAb sample (mAb1, oxidized mAb1, mAb4, and oxidized mAb4) into 57 μL of aqueous buffer (5 mM K_2_HPO_4_, 5 mM KH_2_PO_4_, in H_2_O, pH 7.0). Labeling with deuterium was performed by diluting 3 μL of each mAb sample into 57 μL of deuterated buffer (5 mM K_2_HPO_4_, 5 mM KH_2_PO_4_, in D_2_O, pD 7.0). HDX reactions were maintained at 20°C and allowed to react for 20 s, 100 s, 500 s, 2500 s, and 12,500 s. The exchange reaction was stopped by adding 1:1 (*v/v*) precooled (1°C) quench buffer (200 mM potassium phosphate, 4 M guanidine-HCl, 0.5 M TCEP-HCl, pH 2.5). A total of 75 μL of the quenched HDX reaction sample was injected into the HDX system and passed over a Poroszyme immobilized pepsin 2.1 mm × 30 mm column (Thermo Scientific) at 75 μL/min. The resulting peptic peptides were captured in HPLC mobile phase on a VanGuard C18 trapping 2.1 mm × 5 mm column (Waters) and desalted for 4 mins at 75 μL/min of H_2_O containing 0.1% formic acid. Peptides were then separated on a BEH C18 1.0 mm × 50 mm column (Waters) with an 8 min linear gradient of acetonitrile containing 0.1% formic acid increasing from 8 to 40%. Protein digestion for both non-deuterated and deuterated samples was carried out at 20°C; peptide capture and separation were carried out at 0°C. MS detection was immediately performed after HPLC separation on a Xevo G2-XS QTof mass spectrometer (Waters) running in ESI-positive mode. Each experiment was carried out in triplicate.

### HDX-MS Data Analysis

Peptic peptides were identified based on HPLC separation of non-deuterated protein digest using MS^E^. MS^E^ data was analyzed using ProteinLynx global server 3.0 (Waters). Identified peptides were imported with three parallel non-deuterated experiments into Dynamx 3.0 (Waters). Peptides with sufficient signal intensity and confidence were pooled and used for HDX analysis. Deuterium uptake level (#D) for each peptide at each exchange time point was calculated using Dynamx 3.0 to generate uptake curves for each sample. Data obtained was used to make HDX difference charts in which deuterium labeling for each peptide between the reference and oxidized states was compared.

### SPR

mAbs were captured with anti-Fab antibody that was immobilized (amine coupled) to a CM5 chip. Respective antigen was flowed across two flow cells at concentrations ranging from 0 to 40 nM with a reference subtracted flow cell treated identically but without the mAb, the 0 nM injection was used as a second subtraction resulting in the data being double-reference subtracted. Antigen binding was measured by a Biacore T200 instrument (GE Healthcare Life Sciences). Data was analyzed by comparing antibody-antigen association and dissociation kinetics between reference and oxidized mAbs for mAb1 and mAb4 using Biacore T200 Evaluation Software (GE Healthcare Life Sciences).

## Results and Discussion

### Selective Trp Oxidation of mAb1 and mAb4 Using AAPH

Both mAbs studied in this report have multiple residues that are susceptible to oxidation. mAb1 contains one Trp in CDR2 of heavy chain (HC) (Trp1), one Met in CDR3 of HC (Met1), and three conserved Met in fragment crystallizable (Fc) region (Met2–4) that are susceptible to oxidation. mAb4 contains one Trp in CDR3 of HC (Trp1), one Trp in variable domain of HC (Trp2), one Trp in CDR3 of light chain (LC) (Trp3), and three conserved Met in Fc region (Met1–3) that are susceptible to oxidation. Samples containing high levels of Trp oxidation and minimal levels of Met oxidation are essential to the study of Trp oxidation impact on the structure-function relationship using HDX-MS and SPR.

In this report, susceptible Trp residues of the two mAbs were selectively oxidized by AAPH in the presence of excess free Met. Five days of incubation for mAb1 yielded the following oxidation levels: 86.7% Trp1, 14.5% Met1, 3.0% Met2, 1.2% Met3, and 0.4% Met4 (Table I). Similarly, twenty days of incubation for mAb4 yielded the following oxidation levels: 99.9% Trp1, 85.9% Trp2, 99.8% Trp3, 8.3% Met1, 1.8% Met2, and 5.8% Met3 (Table II). The most abundant Trp oxidation product observed for all oxidized Trp is NFK which has a mass shift of +32 Da relative to the native form (Fig. S1). Other modifications such as deamidation, tyrosine cross-linking, histidine oxidation, and shuffled disulfide bonds were carefully monitored in the oxidized mAb1 and mAb4 samples via the tryptic peptide mapping analysis. No significant modification levels were observed (data not shown). While high levels of oxidation at susceptible Trp residues were achieved for both mAbs, low level of Met oxidation was maintained (<15%). This data demonstrates AAPH with excess free Met incubation is a valid approach to selectively oxidize susceptible Trp residues. A previous report by Folzer *et al*. using the same approach shows similar success of selective oxidation of Trp residues on an IgG1 mAb (37.6% and 82.1%) while maintaining low level of Met oxidation (<11%) (30). The published results and our experimental data support the feasibility and reliability of AAPH treatment in the presence of excess free Met for Trp oxidation related studies.

**Table I:** mAb1 Percent Oxidation Measured by Tryptic Peptide Mapping

	Trp1 (CDR2 HC)	Met1 (CDR3 HC)	Met2 (Fc)	Met3 (Fc)	Met4 (Fc)
Reference	0.2	0.8	1.6	0.8	0.4
AAPH Oxidized	86.7	14.5	3.0	1.2	0.4

**Table II:** mAb4 Percent Oxidation Measured by Tryptic Peptide Mapping

	Trp1 (CDR3 HC)	Trp2 (VH)	Trp3 (CDR3 LC)	Met1 (Fc)	Met2 (Fc)	Met3 (Fc)
Reference	1.4	1.0	3.9	4.3	2.0	3.4
AAPH Oxidized	99.9	85.9	99.8	8.3	1.8	5.8

**Fig. 1:**
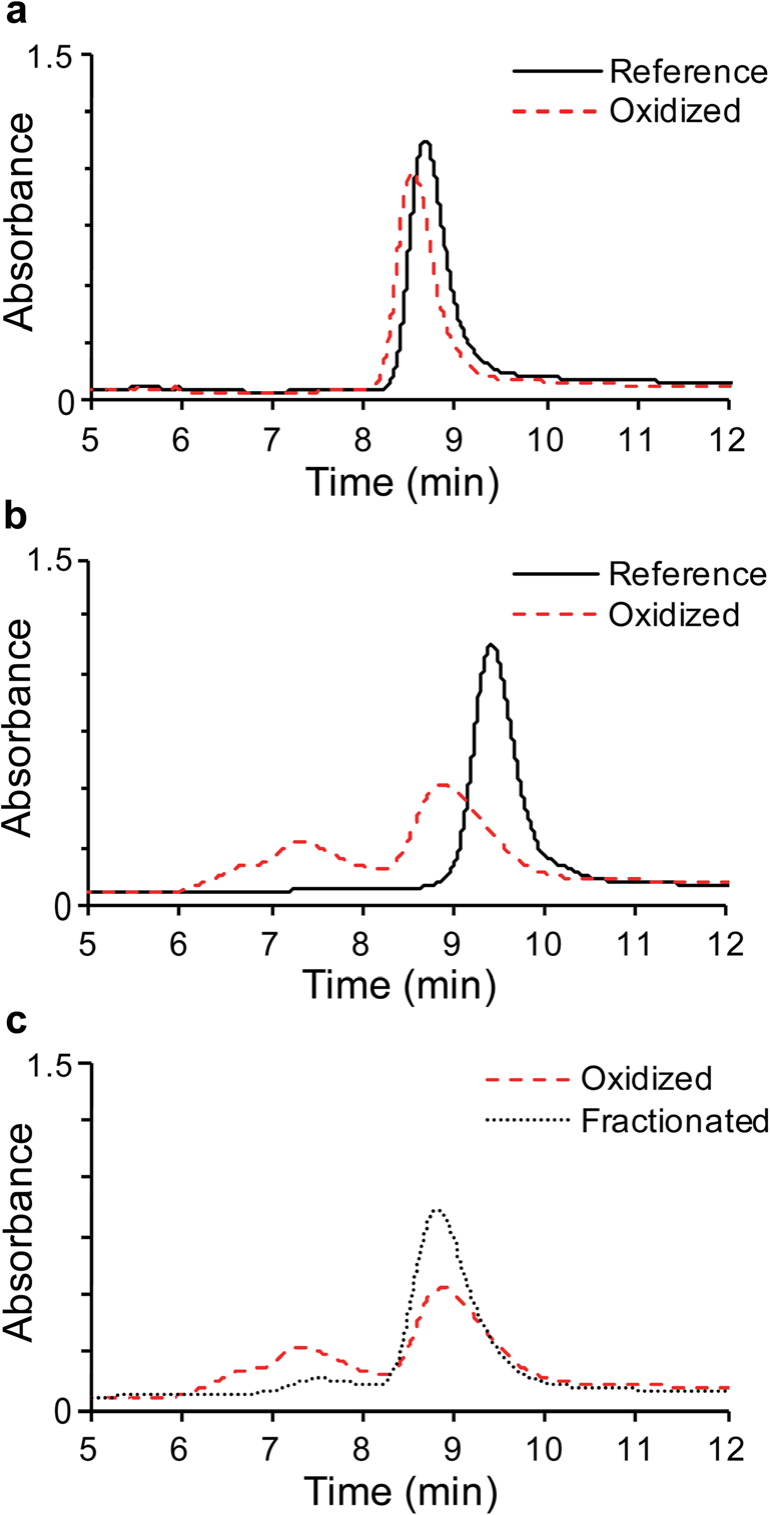
Overlaid SEC chromatograms detected at 220 nm of (**a**) Reference material and oxidized mAb1, (**b**) Reference material and oxidized mAb4, (**c**) Oxidized mAb4 prior to and post (enriched) fraction collection.

However, it has been reported that some mAb oxidation events are associated with increased amount of aggregation. For example Liu at al. showed increased soluble aggregate formation as a function of methionine oxidation in an isolated IgG1 mAb Fc, and Hensel *et al*. made a similar observation with methionine and tryptophan oxidation of an IgG1 mAb (14,25). In our study, the oxidized mAb1 and mAb4 were shown to contain <1% and 38% soluble aggregates by SEC analysis, respectively (Fig. 1a and b). To eliminate interference from soluble aggregates, fractionation by SEC was used to reduce the amount of aggregates in mAb4 to less than 4% (Fig. 1c).

### HDX-MS Results Reveal the Conformational Impact of Trp Oxidation

HDX-MS was performed to characterize the conformational impact of Trp oxidation on mAb1 and mAb4, in which changes in conformation across full length mAbs due to Trp oxidation were measured. Changes could be resolved at the peptide level (5–20 residues). The number of peptides measured by HDX-MS reached 143 peptides (86% total sequence coverage, see Fig. S2) for mAb1 and 140 peptides (88% total sequence coverage, see Fig. S3) for mAb4. These peptides were identified in both the reference and oxidized samples (*n* = 3 for each sample). Peptides that were identified only in the reference sample account for most of the areas of missing coverage for both mAbs. Differences between reference and oxidized samples in deuterium uptake highlight the regions of structural perturbation, at peptide level, due to Trp oxidation.

**Fig. 2:**
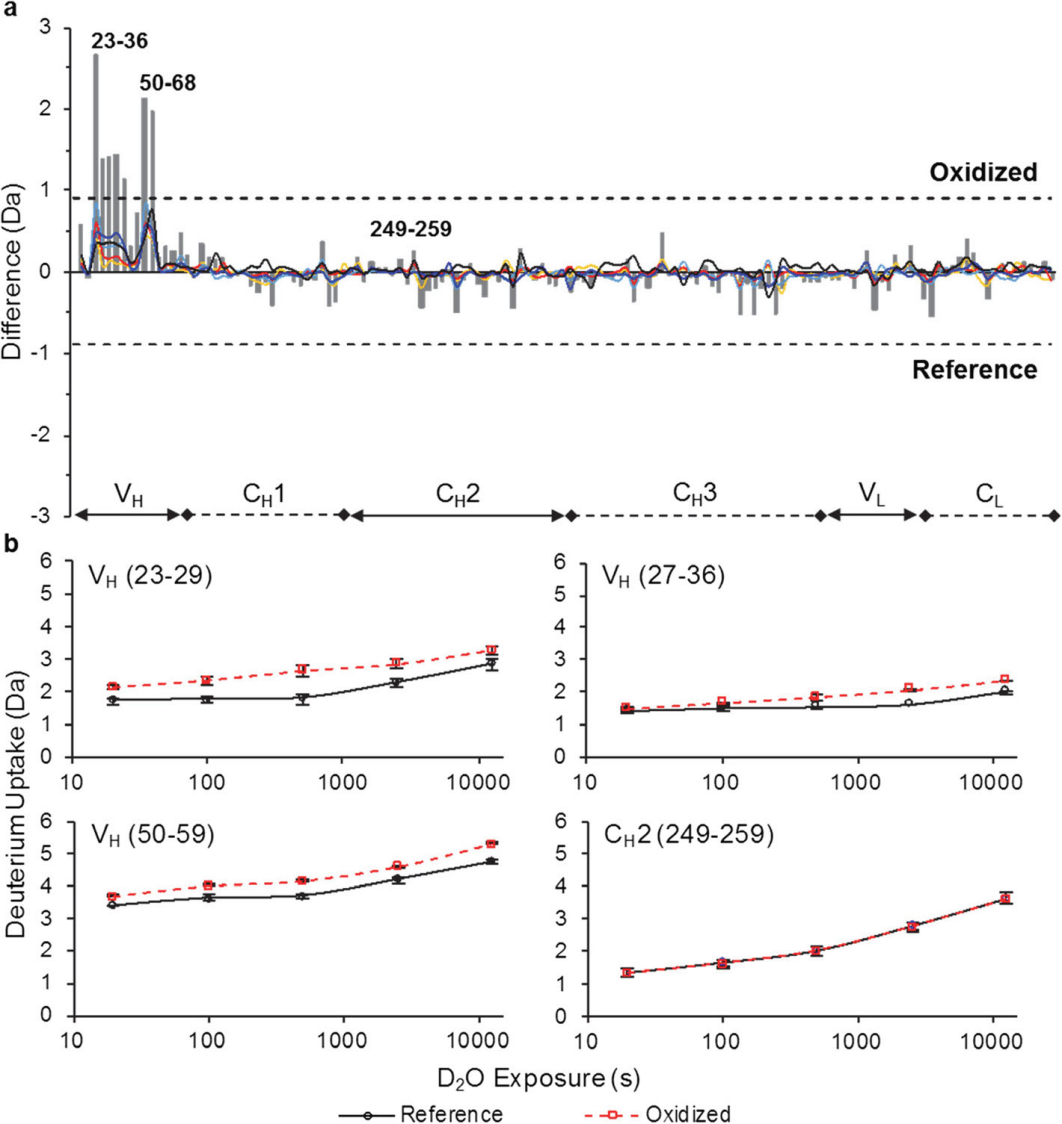
mAb1 HDX-MS (**a**) Summed deuterium uptake differences across 5 exposure times for each peptide. Peptides are arranged on the horizontal axis starting from the N-terminal end of the HC and ending at the C-terminal end of the LC. Bottom plot labels indicate peptide domain locations. The vertical axis is the difference of deuterium uptake between oxidized and reference material. Individual deuterium uptake differences (*∆D*_*t*_ =  # *D*_*t*, *Oxidized*_ –  # *D*_*t*, *Reference*_) are represented as lines: 20 s (gold), 100 s (red), 500 s (cyan), 2500 s (blue), and 12,500 s (black). Summed differences (∑*∆D*_*t*_) are represented as grey bars for each peptide. The dashed lines at ±0.9 Da are the 98% confidence limits for significant summed differences (see Fig. S4). (**b**) Peptide deuterium uptake curves representative of regions labeled in panel **a**. HDX was corrected for Trp oxidation containing peptides (see Fig. S5). 3 × SD error bars for each triplicate measurement are displayed.

Overall, two regions (HC: 23–36 and 50–68) of mAb1 show significantly increased deuterium uptake in oxidized sample compared to the reference (Fig. 2a). Both regions are within the VH domain containing oxidized Trp1. The deuterium uptake curves for overlapping peptides in these two regions show faster deuterium exchange for oxidized mAb1 (Fig. 2b). No other domains of mAb1 showed a significant difference in deuterium uptake. For example, in Fig. 2b, peptide 249–259 of the C_H_2 domain shows similar deuterium uptake profile for reference and oxidized mAb1.

**Fig. 3:**
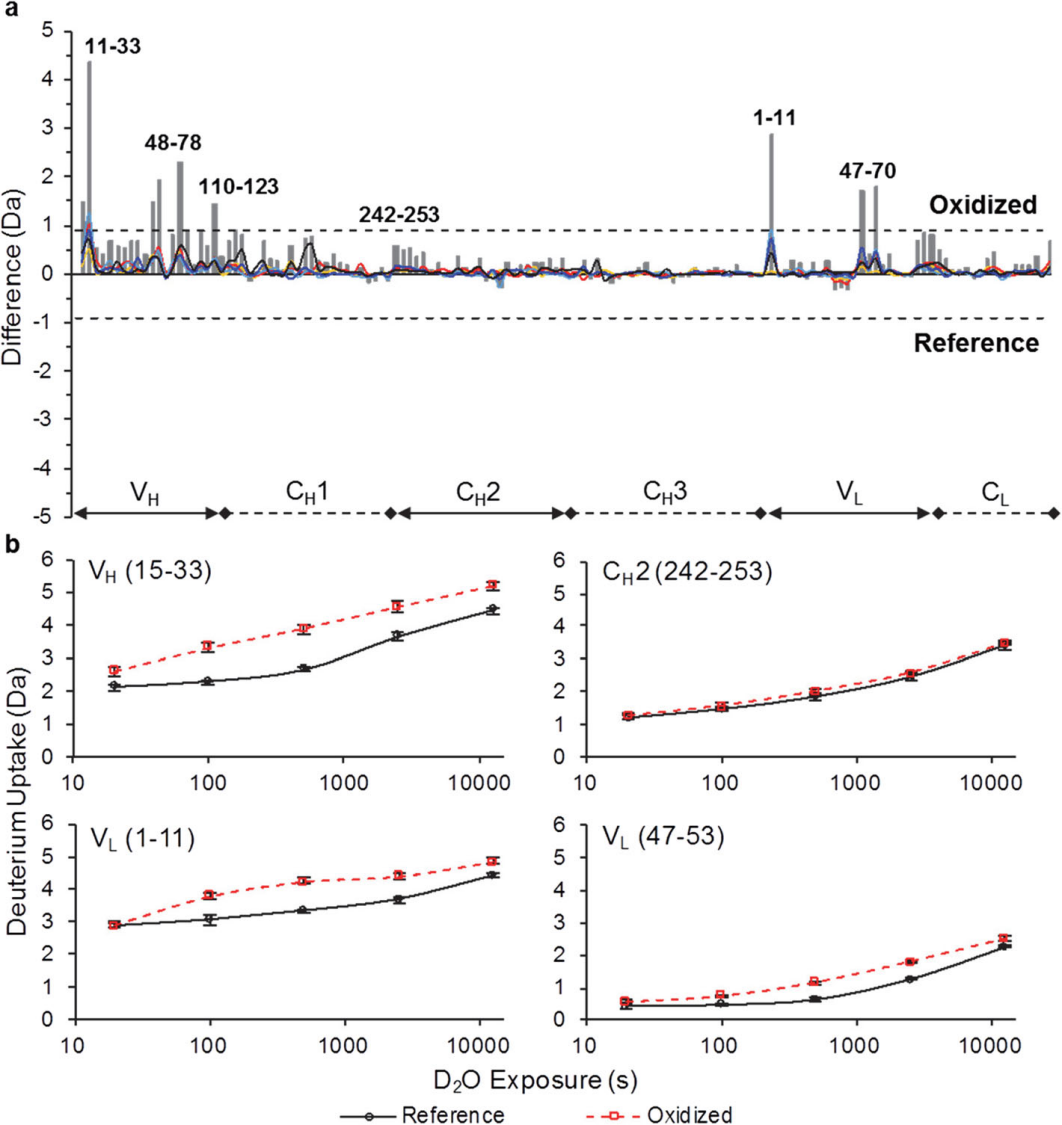
mAb4 HDX-MS (**a**) Summed deuterium uptake differences across 5 exposure times for each peptide. Peptides are arranged on the horizontal axis starting from the N-terminal end of the HC and ending at the C-terminal end of the LC. Bottom plot labels indicate peptide domain locations. The vertical axis is the difference of deuterium uptake between oxidized and reference material. Individual deuterium uptake differences (*∆D*_*t*_ =  # *D*_*t*, *Oxidized*_ –  # *D*_*t*, *Reference*_) are represented as lines: 20 s (gold), 100 s (red), 500 s (cyan), 2500 s (blue), and 12,500 s (black). Summed differences (∑*∆D*_*t*_) are represented as grey bars for each peptide. The dashed lines at ±0.9 Da are the 98% confidence limits for significant summed differences (see Fig. S4). (**b**) Peptide deuterium uptake curves representative of regions labeled in panel **a**. 3 × SD error bars for each triplicate measurement are displayed.

For mAb4, five regions (HC: 11–33, 48–78 and 110–123; LC: 1–11 and 47–70) of mAb4 show significantly increased deuterium uptake in oxidized sample compared to the reference (Fig. 3a). Among them, three regions showing increased flexibility are within VH domain containing oxidized Trp1 and Trp2. Two regions showing increased flexibility are within VL domain containing oxidized Trp3. The deuterium uptake curves for peptides in all five regions show faster deuterium exchange for oxidized mAb4 (Fig. 3b). No other domains of mAb4 showed a significant difference in deuterium uptake, similar to the findings in mAb1.

**Fig. 4:**
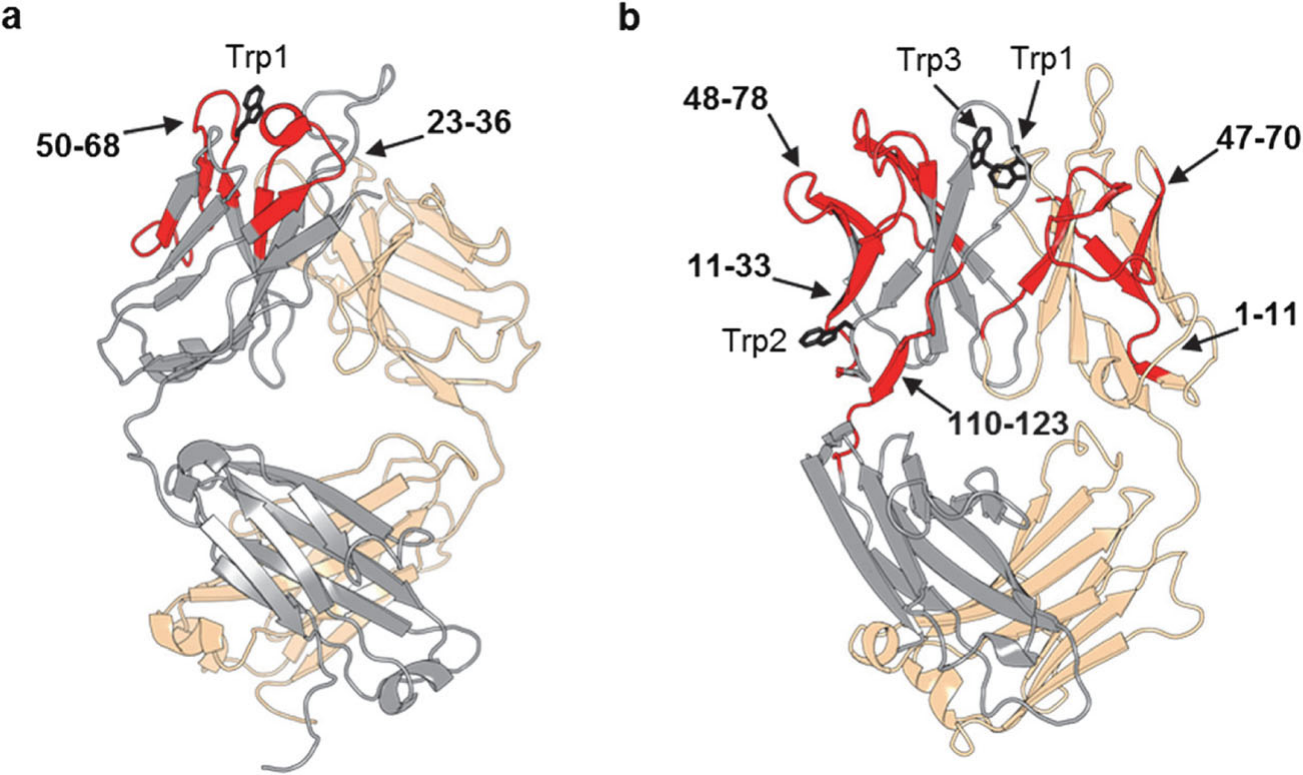
Significant differences in deuterium uptake mapped onto homology structure of Fab. HC (grey), LC (wheat), oxidized Trp (black), and increased HDX (red). (**a**) mAb1 (PDB 1HZH) mapped increased HDX of oxidized in labeled regions of HC: 23–36 and 50–68. (**b**) mAb4 (PDB 5DK3) mapped increased HDX of oxidized in labeled regions of HC: 11–33, 48–78 and 110–123; LC: 1–11 and 47–70.

HDX-MS results also revealed Trp oxidation increased flexibility in nearby regions to different extents for mAb1 and mAb4. Oxidation of Trp1 in CDR2 of HC of mAb1 increased flexibility of the region covering both CDR2 and CDR1 of HC (23–36 and 50–68). This is not surprising since the homology model for an IgG1 Fab (PDB 1HZH) indicates these two regions are in proximity of each other in the 3-D structure (Fig. 4a). Modifications such as adding two oxygen atoms to the side chain of Trp1 (shown as black sticks in Fig. 4a) will increase spatial occupancy and decrease the hydrophobicity of the Trp side chain. This chemical change to Trp1 not only destabilizes CDR2 of HC, but also destabilizes CDR1 of HC. Conceptually, CDR3 of HC is likely to be affected too, since it is in proximity of both conformationally perturbed regions. Unfortunately, it is in an area of missing coverage, thus no experimental evidence was obtained (Fig. S2). In addition, CDR1 and CDR3 of LC are in areas of missing coverage, but their conformation could be impacted by the Trp1 oxidation too.

For mAb4, the three oxidized Trp residues (Trp1, Trp2, and Trp3) are located in CDR3 of HC, variable region of HC, and CDR3 of LC, respectively. HDX-MS data show increased flexibility in regions dispersed across variable regions of heavy chain and light chains (VH and VL). The homology model for an IgG4 Fab (PDB 5DK3) indicates these regions are close to the Trp oxidation sites (Fig. 4b). Region 11–33 spans CDR1 of HC, with the N-terminus close to Trp2 and C-terminus close to both Trp1 and Trp3. Region 48–78 spans CDR2 of HC, near destabilized region 11–33, and is close to both Trp1 and Trp3. Region 110–123 of HC is near Trp2 with the N-terminus close to CDR3 of HC containing Trp1. N-terminus of region 1–11 of LC is near Trp3. Region 47–70 spans CDR2 of LC and is near Trp1 and destabilized region 110–123 on HC. All the VH and VL regions with increased flexibility upon Trp oxidation are either in proximity of oxidation site(s) or in proximity of other destabilized regions. Oxidized Trp1 and Trp3 of CDR3 of HC and LC, respectively, are in proximity of conformationally perturbed regions of HC (11–33 and 48–78) and LC (1–11 and 47–70). The pattern of conformational destabilization suggests that the CDR3 region conformations are also impacted by oxidation of Trp1 or Trp3. However, since they are in an area of missing coverage, no experimental evidence could be obtained (Fig. S3). Also, the N-terminus of HC that contains oxidized Trp2 is in an area of missing coverage. Conformation in this region is likely to be impacted as well due to destabilized neighboring regions of HC (11–33 and 110–123).

Comparing HDX-MS results of mAb4 to mAb1, the former shows a more dispersed increase of flexibility across both VH and VL. This is not surprising considering there are three Trp oxidation sites in the two domains of mAb4 *vs* only one in mAb1. This data demonstrates Trp oxidation destabilized nearby regions and caused increasing flexibility in protein conformation. The treatment by AAPH and excess free Met provided the analytical probe to uncover the conformational impact of Trp oxidation. Minimal Met oxidation along with the absence of other modifications were essential in maintaining the selectivity of the study that was focused on Trp oxidation, as other modifications may have structural and/or functional impact on mAbs as well. For example, Met oxidation in Fc region of mAbs may cause conformational changes in the C_H_2 domain, contributing to an aggregation hotspot in region FLFPPKPKDTLM (36). Minimizing Met oxidation during AAPH treatment allowed us to avoid such changes and probe the impact of Trp oxidation alone. The fact that deuterium uptake curves show little difference between oxidized and reference samples in the above mentioned region for mAb1 (HC 249–259) and for mAb4 (HC 242–253) further demonstrates the selectivity of AAPH treatment for Trp oxidation with minimal Met oxidation.

The HDX-MS results highlight the conformational impact of Trp oxidation on VH and VL of mAb1 and mAb4. Most of the Trp residues are buried in hydrophobic cores and are not susceptible to oxidation. However, Trp provides unique touch points for protein-protein interactions hence they are frequently found in the CDRs of mAbs. Trp residues in CDRs not only play important roles in antigen binding, but also in maintaining conformational stability of CDRs. CDRs carry conformational flexibility, which contributes to their functionality as paratopes for antigen binding. With underlying flexibility and exposure, Trp in CDRs are unable to pack tightly as they would in a hydrophobic core. Even with the lack of packing for Trps in the CDRs, their oxidation disrupts native conformation of VH and VL. Such destabilization can alter CDR’s functionality and impact long term stability.

### SPR Results Indicate Disrupted Antigen Binding Caused by Trp Oxidation in CDRs

SPR by Biacore was used to characterize the impact of Trp oxidation on antigen binding for mAb1 and mAb4. Kinetic association and dissociation rates of antigen with mAb were measured by SPR. Capture of reference mAbs and oxidized mAbs were compared and shown to be similar (see Fig. S6), which indicates structural perturbations by oxidation in variable domains did not disrupt the capture of oxidized mAb by anti-Fab for SPR analysis.

**Fig. 5:**
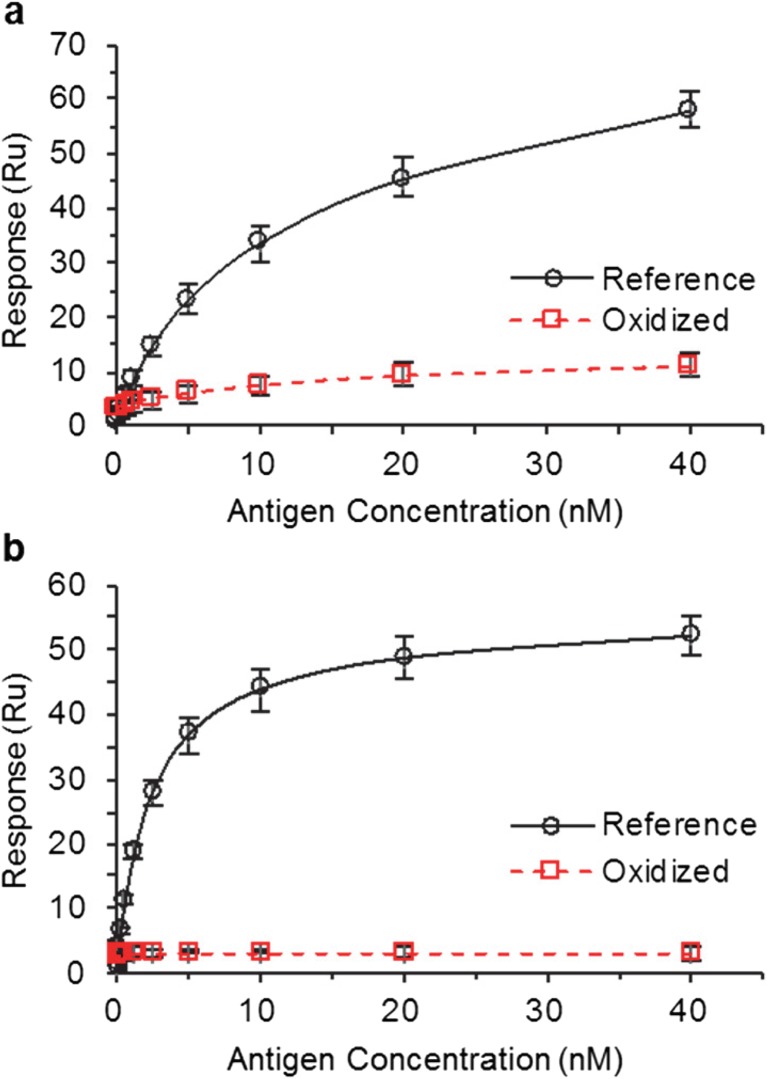
SPR equilibrium binding response on vertical axes *vs*. antigen concentration on horizontal axes. (**a**) Reference standard and oxidized mAb1. Oxidized sample showed 19.4 ± 5.7% relative antigen binding when comparing equilibrium binding response at 40 nM antigen concentration. (**b**) Reference standard and oxidized mAb4. Oxidized sample showed 6.1 ± 6.0% relative antigen binding when comparing equilibrium binding response at 40 nM antigen concentration.

Taking a window of response at an equilibrium binding point for each antigen concentration respective to each mAb allowed us to plot response *versus* antigen concentration (see Fig. S7 and Fig. S8). At binding equilibrium of the highest antigen concentration (40 nM), oxidized mAb1 showed 19.4% relative antigen binding compared to reference standard, as calculated by taking a ratio of the response for the oxidized material to the response for the reference standard (Fig. 5a). These results show oxidation of Trp1 in CDR2 of HC disrupts antigen binding for mAb1. Kinetic constants for oxidized mAb1 cannot be reliably calculated due to the lack of antigen binding response. Kinetic constants for reference mAb1 are in the expected range from previous development measurements (see Table S1). Each of the concentrations was injected in triplicate; the error associated with the binding equilibrium at the highest antigen concentration for mAb1 is ±5.7%. Considering there is approximately 15% of unmodified mAb1 in the oxidized sample (Table I), the 19.4% relative percent antigen binding was likely contributed by the unmodified molecules.

At binding equilibrium of the highest antigen concentration (40 nM), oxidized mAb4 showed 6.1% relative antigen binding compared to reference standard (Fig. 5b). These results show oxidation of Trp1 in CDR3 of HC, Trp2 in VH, and Trp3 in CDR3 of LC significantly disrupt mAb interaction with antigen. Again, the kinetic constants for oxidized mAb4 cannot be reliably calculated due to the lack of antigen binding response. Kinetics for mAb4 reference standard are in the expected range from previous development measurements (see Table S1). The error associated with the binding equilibrium at the highest antigen concentration for mAb4 is ±6.0%.

The disrupted antigen binding for mAb1 can be explained by the local conformational perturbation exerted by Trp oxidation which increased the flexibility of CDR2 of HC and CDR1 of HC. Also, it is important to consider the chemical change associated with Trp oxidation. Addition of two oxygen atoms to a Trp side chain drastically changes the nature of a Trp side chain, which consequently changes the native chemical properties of Trp. If Trp is a strong contact point in the antigen-antibody interface, then a combination of increased flexibility across two CDRs of the HC and the associated chemical change in Trp severely disrupted antigen-mAb1 interaction.

Similarly, the disrupted antigen binding for mAb4 can be explained by the dispersed conformational perturbations from Trp oxidation, which increased flexibility across both VH and VL. With two Trp residues lying within the CDRs near each other and four oxygen atoms added in that space, the chemical environment changes significantly. The impact on antigen binding caused by Trp oxidation with the associated structural perturbations (increased flexibility) and chemical changes are similar for mAb1 and mAb4.

## Conclusion

AAPH treatment in the presence of excess free Met is a suitable approach to selectively oxidize Trp for site specific degradation studies, such as structure-function relationship studies concerning Trp oxidation. Oxidation of Trp in CDRs of mAbs significantly disrupts antigen binding as shown for the IgG1 and IgG4 mAbs used in this study. Oxidation of Trp in CDRs of mAbs alters conformation in variable domains to different extents as shown in mAb1 and mAb4. Chemical modification brought by Trp oxidation can lead to undesirable physical changes in mAbs such as increased propensity for aggregation and loss of potency. The increased flexibility in the CDRs containing oxidized Trp resulted in the loss of antigen binding activity for the studied IgG1 and IgG4 mAbs, which is also highly applicable to other mAbs containing CDRs with Trp residues. Additional studies can be performed to further explore the correlation between increased flexibility with protein stability, which could be a long term impact stemming from Trp oxidation. In conclusion, this study provides a highly effective tool for understanding the structure function relationships for CDR Trp oxidation without the interference from other degradations. CDR Trp oxidation should be carefully monitored during process development of mAbs. If susceptible Trp residues are found at an impactful rate in well-controlled products, it should be considered in critical quality attribute assessments in which a comprehensive understanding of degradation pathways and impact on product quality are essential in order to establish appropriate control strategies for therapeutic proteins.

### ACKNOWLEDGEMENTS AND DISCLOSURES

The authors would like to thank Dr. David Weis at the University of Kansas for his support during this study, and Dr. Reb Russell for encouragement and support.

## Electronic supplementary material

ESM 1 (DOCX 529 kb)

## Abbreviations

AAPH: 2,2′-Azobis(2-amidinopropane) dihydrochloride
CDRs: Complementarity-determining regions
HDX-MS: Hydrogen-deuterium exchange mass spectrometry
mAbs: Monoclonal antibodies
Met: Methionine
SPR: Surface plasmon resonance
Trp: Tryptophan
