# Supplementary material for: Impact of Tryptophan Oxidation in Complementarity-Determining Regions of Two Monoclonal Antibodies on Structure-Function Characterized by Hydrogen-Deuterium Exchange Mass Spectrometry and Surface Plasmon Resonance
Source: Pharm Res. 2018 Dec 10;36(1):24. doi: 10.1007/s11095-018-2545-8 (PMC6290686; doi:10.1007/s11095-018-2545-8)
Supplement: Supplementary file 1 — (DOCX 529 kb) [file 11095_2018_2545_MOESM1_ESM.docx]

**Supporting Information**

**Impact of Tryptophan Oxidation in Complementarity-Determining Regions of two Monoclonal Antibodies on Structure-Function Characterized by Hydrogen-Deuterium Exchange Mass Spectrometry and Surface Plasmon Resonance**

Tyler Hageman^^[[1]](#footnote-1)^,^[[2]](#footnote-2)^^; Hui Wei^2^; Patrick Kuehne^2^; Jinmei Fu^2^; Richard Ludwig^2^; Li Tao^2^; Anthony Leone^2^, Marcel Zocher^2^; Tapan K. Das^2^

**Supplemental Figure S1** Tryptophan oxidation products

**
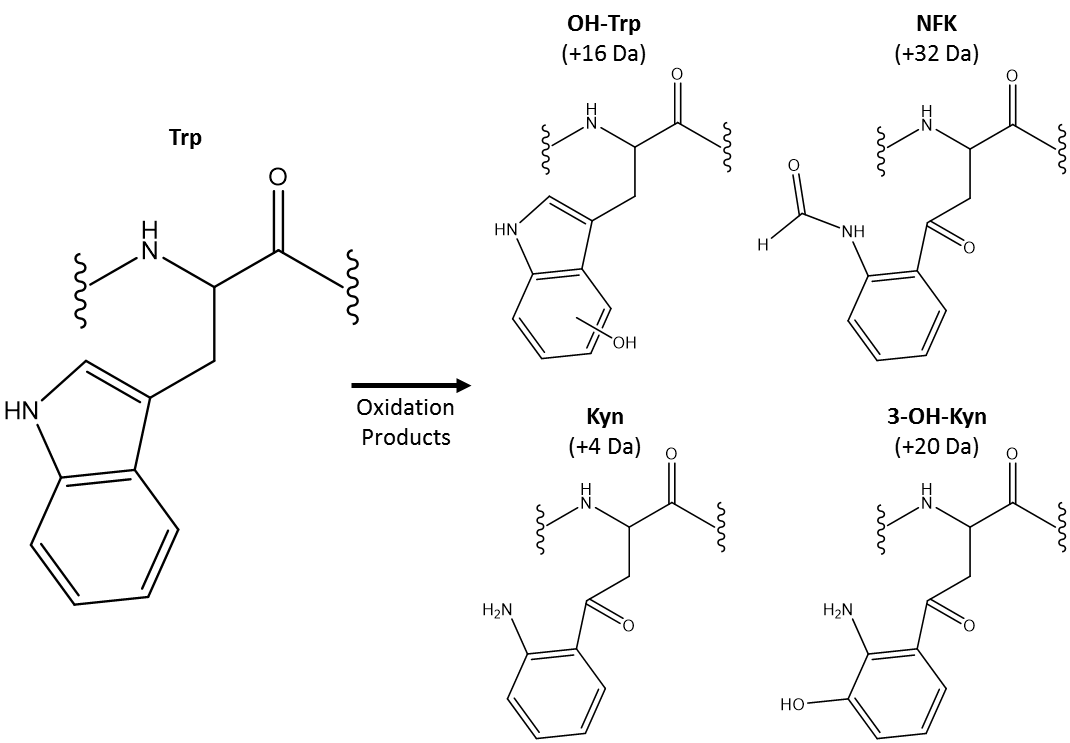
**

**Supplemental Figure S2** HX-MS measured peptides sequence coverage map for mAb1 reference and oxidized

**Supplemental Figure S3** HX-MS measured peptides sequence coverage map for mAb4 reference and oxidized

**Supplemental Figure S4** Summed deuterium uptake difference plots construction

Calculating significance and plotting summed deuterium uptake difference plots followed the algorithm by Houde, D.; Berkowitz, S.A.; Engen, J.R. *J. Pharm Sci*. 2011, 100, 2071-2086.

mAb1 = 143 peptides; 2 states (6 time points (0 s, 20 s, 100 s, 500 s, 2500 s, 12500 s); n=3 all)

mAb4 = 140 peptides; 2 states (6 time points (0 s, 20 s, 100 s, 500 s, 2500 s, 12500 s); n=3 all)

3,432 mean #D values with 3,432 SDs

$$\left( 3 \times{SD}_{Avg} \right)=\pm0.1 Da$$

$$Standard error of \#D mean= \frac{0.1 Da}{\sqrt{3}}= \pm0.058 Da$$

$$Propagation oferror for \sum{\Delta D}_{t}= \sqrt{5 \times{(0.058)}^{2}}=\pm0.13 Da$$

$$98\% confidene limit of\sum{\Delta D}_{t}= 0.13 Da \times{6.965}_{t_{98\%, df = 2}}= \pm0.9 Da$$

**Supplemental Figure S5** HX correction for monitored oxidized peptides of mAb1 reference and oxidized

Trp oxidized peptides elute earlier in reverse-phase chromatography than unmodified Trp containing peptides. Change in retention time will impact back exchange for each peptide. To correct for this difference in back exchange we measured the effect of retention time shift on back exchange. In our study two Trp oxidized peptides HX rates were measured and compared to reference unmodified peptides. To calculate the back exchange correction alternative gradients were used to elute the unmodified peptide at the same retention time as the Trp oxidized peptides. Deuterium uptake at 500 s exposure time was compared for these peptides at initial retention time and alternative retention time. The back exchange correction for each peptide was measured to be 0.1 Da. 0.1 Da was subtracted from each #D mean value at each time point across both measured Trp oxidized peptides.

**Supplemental Figure S6** Raw anti-Fab capture loading sensorgrams for SPR

Oxidized mAbs and reference material were captured using immobilized anti-Fab antibody. Conformational perturbations to variable regions from Trp oxidation didn’t disrupt anti-Fab capture for SPR measurements.

**Supplemental Figure S7** SPR sensorgrams for mAb1 reference and oxidized

**Supplemental Figure S8** SPR sensorgrams for mAb4 reference and oxidized

**Supplemental Table S1** SPR calculated K_D_ for mAb1 and mAb4

|  | K_D_ |
| --- | --- |
| mAb1 reference | 2.04 nM |
| mAb4 reference | 0.14 nM |

These values are similar to previously measured values of mAb1 stock material (1.92 nM) and mAb4 stock material (0.15 nM). Measured response of oxidized mAbs was too low to calculate reliable K_D_.

1. Department of Chemistry, University of Kansas, Lawrence, KS, USA [↑](#footnote-ref-1)
2. Biologics Development, Bristol-Myers Squibb, Pennington, NJ, USA [↑](#footnote-ref-2)
